# Supplementary material for: Differentiation and Growth-Arrest-Related lncRNA (DAGAR): Initial Characterization in Human Smooth Muscle and Fibroblast Cells
Source: Int J Mol Sci. 2024 Aug 31;25(17):9497. doi: 10.3390/ijms25179497 (PMC11394763; doi:10.3390/ijms25179497)
Supplement: Supplementary file 1 [file ijms-25-09497-s001.zip › ijms-3152544-supplementary.pdf]

Supplementary Materials

Supplementary Figure Legends

Figure S1. A) lncRNA screening strategy in hPASMC differentiation. Schematic overview of human Pulmonary Artery Smooth Muscle Cells (hPASMC) differentiation by cell-to-cell contact. Cells at 70-80% confluence (D0) show high rates of proliferation and migration (left). At this stage, cells express proliferation markers, with low SMC marker gene expression. When SMC reach 100% confluence (D2), they start to align and differentiate, with a concomitant decrease in proliferation and migration. Four days after reaching confluence, SMC become fully differentiated (D6), marker gene expression is induced and accompanied by an increase in contractile capabilities. At this stage, migration is abrogated, and cells stop proliferating, as they exit the cell cycle in G0/G1 and remain quiescent. **B)** RNA sequencing in human pulmonary artery smooth muscle cells (hPASMCs) at D0 and D2. The figure highlights regulated transcripts in D2 compared to D0. **C)** Validation of differentiation state in RNA sequencing data. Transcripts known to be either down (left) or upegulated (right) at the beginning of the differentiation process in SMCs. **D)** Six lncRNA candidate expression validation by RT-qPCR. D0 = hPASMCs at 70-80% confluence. D2 = hPASMCs at 100% confluence. D6 = hPASMCs 4 days after 100% confluence. hPAEC = human pulmonary artery endothelial cells at 100% confluence. Note that the selected candidate (lncRNA4 or *DAGAR*) is strongly induced after reaching 100% confluence in hPASMCs and is not expressed in hPAEC.

Figure S2. A) RT-qPCR of *DAGAR*-1 and *DAGAR*-2 at D0 and D6 of SMC differentiation **B)** RNA agarose gel after OligodT pulldown showing total, polyadenylated (Poly A+) and not polyadenylated (Poly A-) fractions (Left). *DAGAR* expression relative to Total RNA fraction analyzed by RT-qPCR (right). **C)** RT-qPCR of *DAGAR*-1 and *DAGAR*-2 after siDAGAR compared to scrambled siPool control (siCT). *p<0.05, **p<0.01.

Figure S3. A) mRNA and protein expression of YTHDF2 in proliferative MRC5 after siYTHDF2 compared to scrambled siPool control (siCT). **B)** mRNA and protein expression of YTHDF2 in hPASMC D2 after siYTHDF2 compared to scrambled siPool control (siCT)

Figure S4. Reactome Pathways enrichment analysis from proteins co-precipitated with *DAGAR* (DAGAR RIP). Top 30 enriched pathways ranked by Gene Ratio. For a complete list of enriched pathways refer to Supplementary table 5. ***p<0.001, ****p<0.0001.

Figure S1

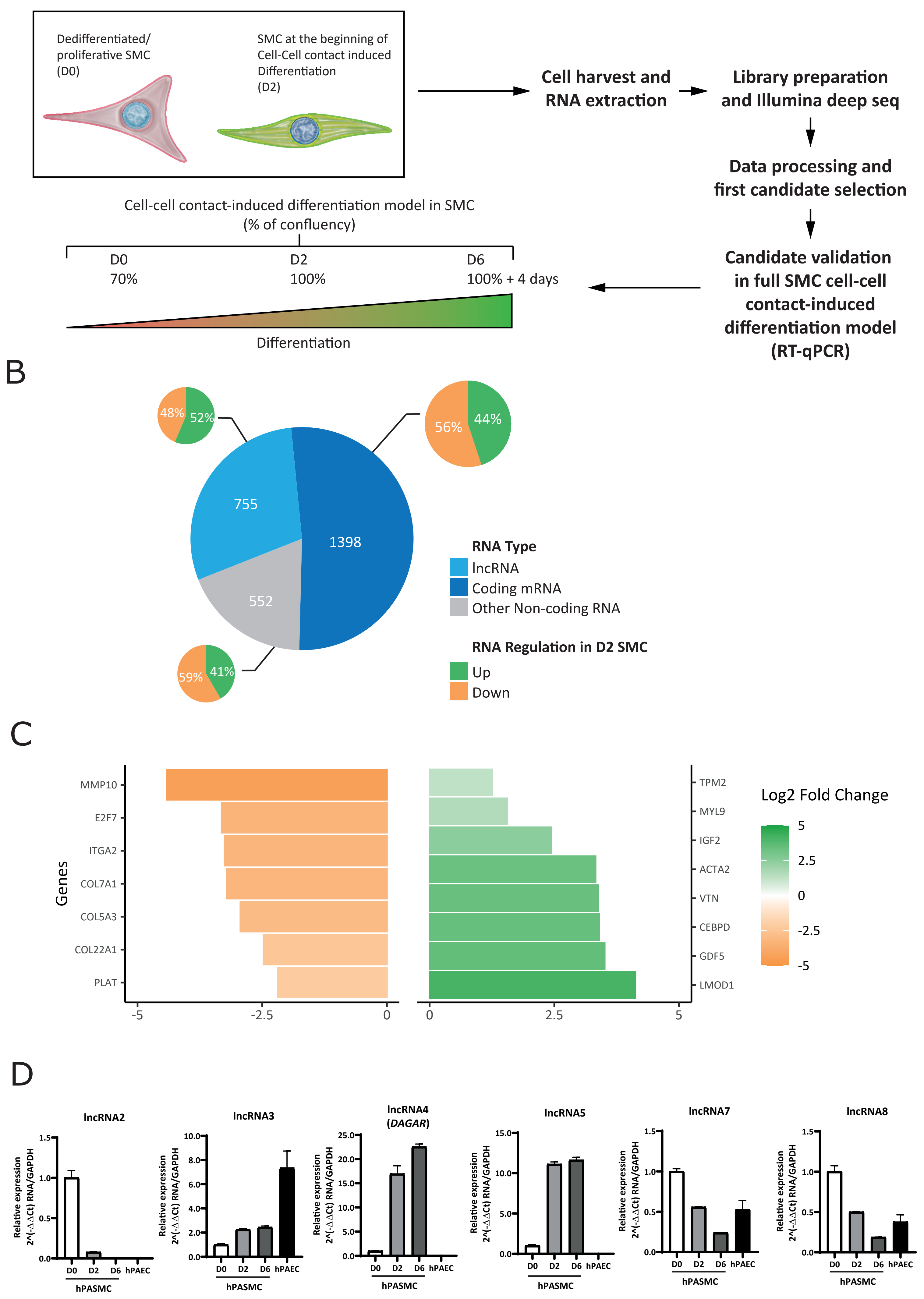
A
Dedifferentiated/ proliferative SMC (D0)
SMC at the beginning of Cell-Cell contact induced Differentiation (D2)
Cell harvest and RNA extraction
Library preparation and Illumina deep seq
Data processing and first candidate selection
Candidate validation in full SMC cell-cell contact-induced differentiation model (RT-qPCR)
Cell-cell contact-induced differentiation model in SMC (% of confluency)
D0 70%
D2 100%
D6 100% + 4 days
Differentiation
B
48%
52%
56%
44%
755
1398
552
59%
41%
RNA Type
lncRNA
Coding mRNA
Other Non-coding RNA
RNA Regulation in D2 SMC
Up
Down
C
Genes
MMP10
E2F7
ITGA2
COL7A1
COL5A3
COL22A1
PLAT
TPM2
MYL9
IGF2
ACTA2
VTN
CEBPD
GDF5
LMOD1
-5
-2.5
0
2.5
5
Log2 Fold Change
D
lncRNA2
lncRNA3
lncRNA4 (DAGAR)
lncRNA5
lncRNA7
lncRNA8
Relative expression 2^(-ΔΔCt) RNA/GAPDH
D0 D2 D6 hPAEC
hPASMC

Figure S2

A

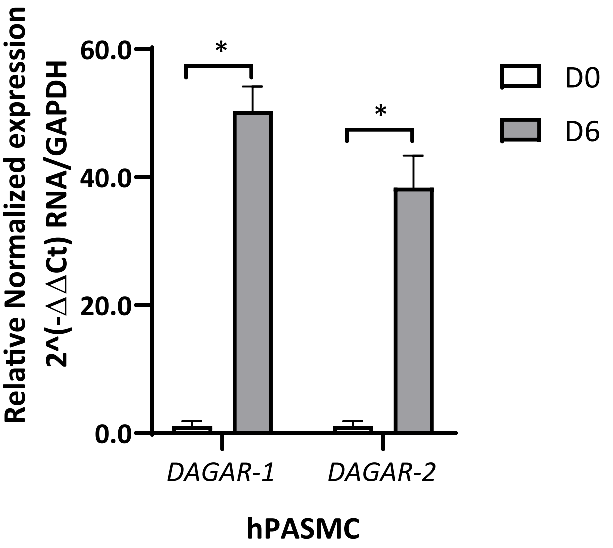

B

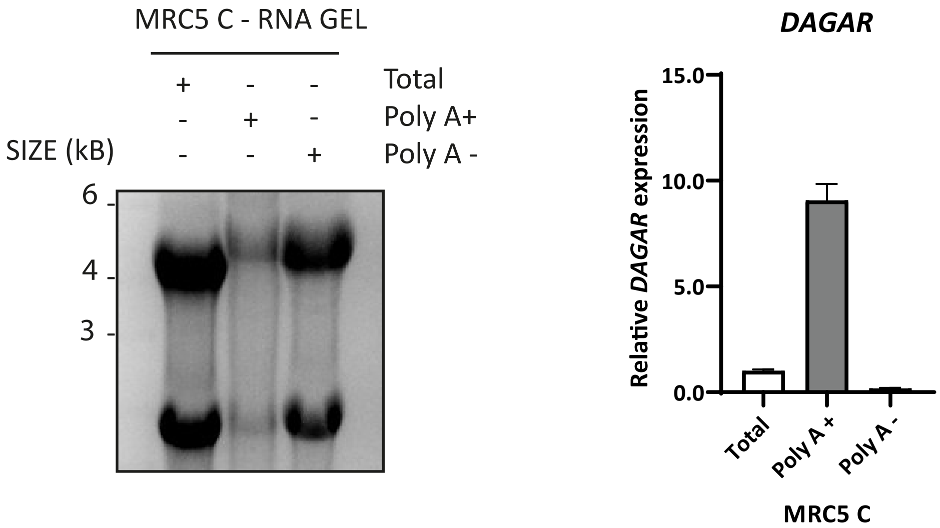

C

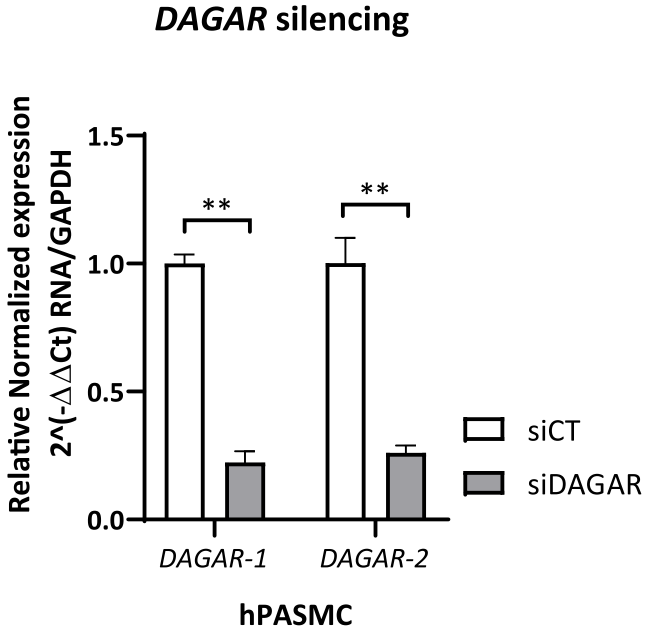

Figure S3

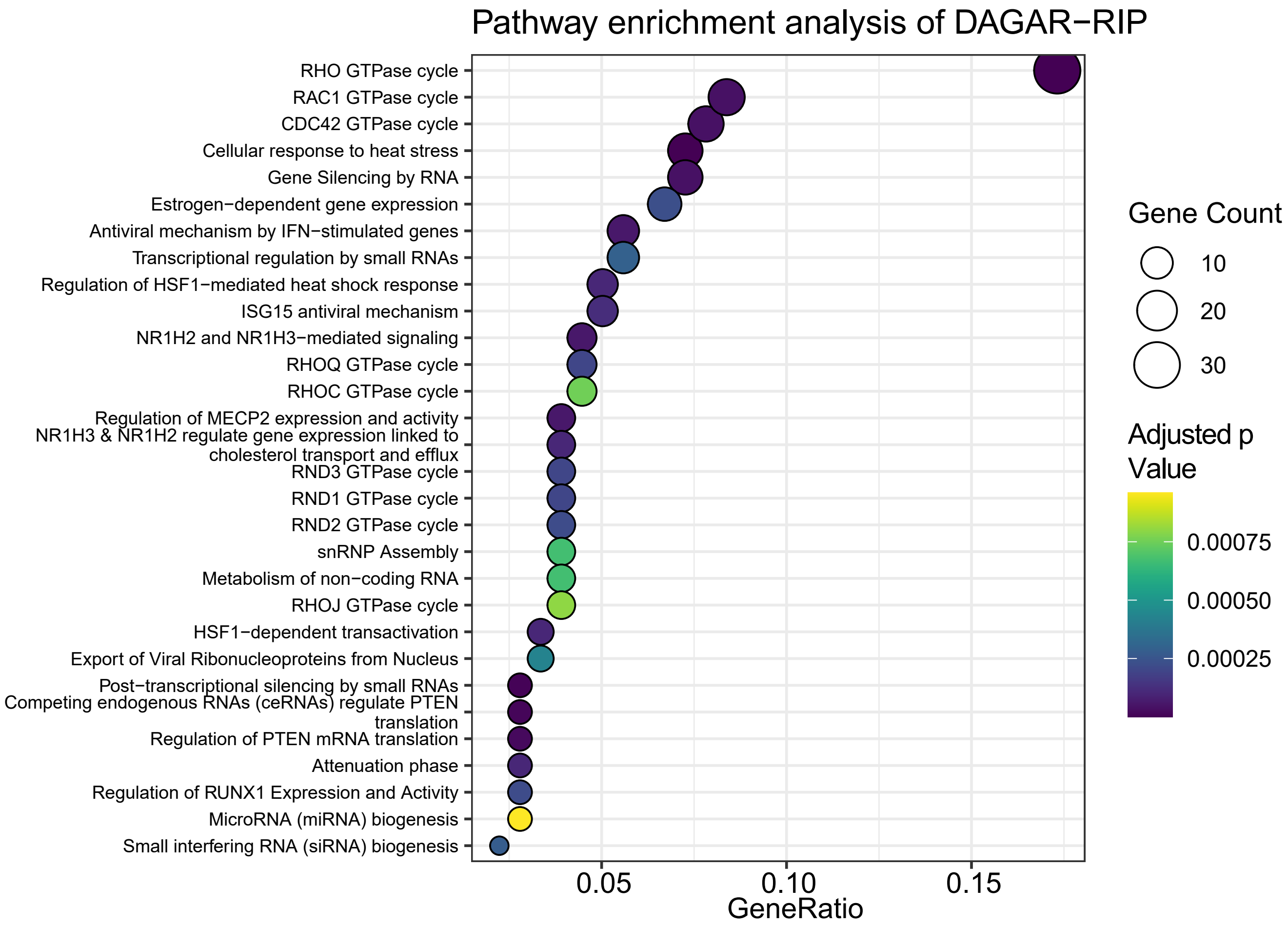

Pathway enrichment analysis of DAGAR−RIP
RHO GTPase cycle
RAC1 GTPase cycle
CDC42 GTPase cycle
Cellular response to heat stress
Gene Silencing by RNA
Estrogen−dependent gene expression
Antiviral mechanism by IFN−stimulated genes
Transcriptional regulation by small RNAs
Regulation of HSF1−mediated heat shock response
ISG15 antiviral mechanism
NR1H2 and NR1H3−mediated signaling
RHOQ GTPase cycle
RHOC GTPase cycle
Regulation of MECP2 expression and activity
NR1H3 & NR1H2 regulate gene expression linked to cholesterol transport and efflux
RND3 GTPase cycle
RND1 GTPase cycle
RND2 GTPase cycle
snRNP Assembly
Metabolism of non−coding RNA
RHOJ GTPase cycle
HSF1−dependent transactivation
Export of Viral Ribonucleoproteins from Nucleus
Post−transcriptional silencing by small RNAs
Competing endogenous RNAs (ceRNAs) regulate PTEN translation
Regulation of PTEN mRNA translation
Attenuation phase
Regulation of RUNX1 Expression and Activity
MicroRNA (miRNA) biogenesis
Small interfering RNA (siRNA) biogenesis
0.05
0.10
0.15
GeneRatio
Gene Count
10
20
30
Adjusted p Value
0.00075
0.00050
0.00025

Figure S4

A

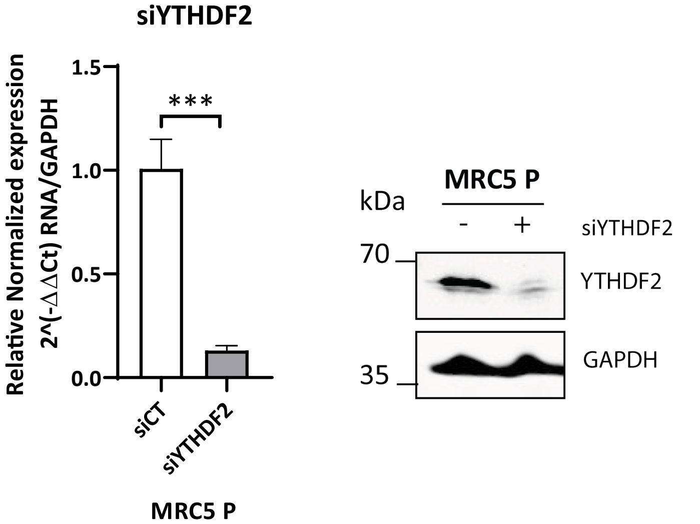
siYTHDF2
Relative Normalized expression 2^(-ΔΔCt) RNA/GAPDH
1.5
1.0
0.5
0.0
***
siCT
siYTHDF2
MRC5 P
kDa
MRC5 P
-
+
siYTHDF2
70
35
YTHDF2
GAPDH

B

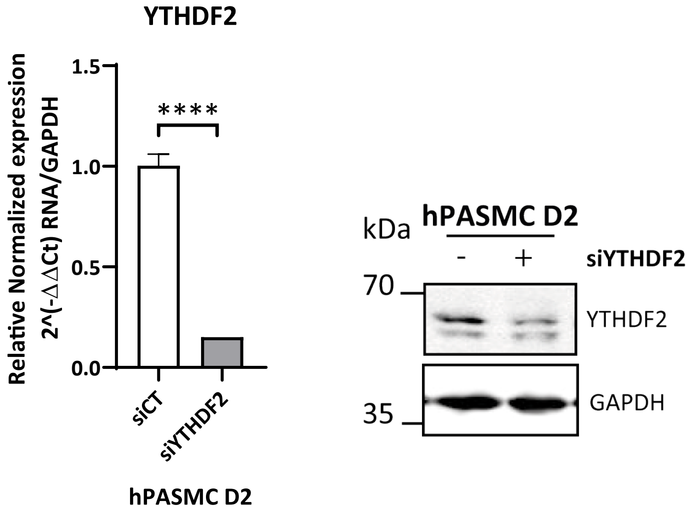
YTHDF2
Relative Normalized expression 2^(-ΔΔCt) RNA/GAPDH
1.5
1.0
0.5
0.0
****
siCT
siYTHDF2
hPASMC D2
kDa
hPASMC D2
-
+
siYTHDF2
70
35
YTHDF2
GAPDH
