# Supplementary material for: Differentiation and Growth-Arrest-Related lncRNA (DAGAR): Initial Characterization in Human Smooth Muscle and Fibroblast Cells
Source: Int J Mol Sci. 2024 Aug 31;25(17):9497. doi: 10.3390/ijms25179497 (PMC11394763; doi:10.3390/ijms25179497)
Supplement: Supplementary file 1 [file ijms-25-09497-s001.zip › Table S1- Primers used.pdf]

Primers used

Table S1. Primers table. The sequences of each primer used in this work are presented. In the column GENE (Observation) the gene name is detailed and observations about the amplicon are between parentheses. The Forward and Reverse sequences are detailed in the remaining columns.

**GENE (Observation)**	**Primer Forward 5' - 3'**	**Primer Reverse 5' - 3'**
CNN1	CACGACATTTTTGAGGCCAA	TTTCCTTTCGTCTTCGCCAT
DAGAR	GGGAAATTCCTGGGATGCCA	TGGGAGCTCCACCTCTAGAC
DAGAR (END)	TCCATTTTCATCCTGCTGTG	CTGGAGTGCAGTTGCATGAT
DAGAR (START)	AGAATCCACATGGAGAACAGTG	AGGAGGTTCCAGCCAGTTTT
DAGAR (MIDDLE)	CACATGGGCTACATCTGCAC	CTTTGCCTTCTGCCATGAAT
DAGAR (NB 1)	GAGCTCCCATTGTGGGCAGAGGGAC	TATAGGCATCGTATTTTAATAAACAG
DAGAR (NB 2)	TCTGAAAACTGGCTGGAACC	CAATGGGAGCTCCACCTCTA
DAGAR1	ATGGAGAACAGTGGGAGGAG	ACCCAGTTTGTGGGACTTTG
DAGAR2	ATGGAGAACAGTGGGAGGAG	TCTGCAGCTCCTGTCTTCAA
GAPDH	GTCTCCTCTGACTTCAACAGCG	ACCACCCTGTTGCTGTAGCCAA
Ki67	GCAGCCTTAACTGTGACACTTGC	GCCACCGTGCCCTGG
MYOCD	GCACCAAGCTCAGCTTAAGGA	TGGGAGTGGGCCTGGTTT
TAGLN (Sm22a)	GGAAGCCTTCTTTCCCCAGA	TCCAGCTCCTCGTCATACTTCTT
YTHDF2	AGGGACAAAAGCCTCCGCC	CCTTGACCTTTTGGTCTCTGCT
